# Wish to die and healthcare use in older people: cross-sectional findings from The Irish Longitudinal Study on Ageing (TILDA)

**DOI:** 10.1007/s10389-025-02647-2

**Published:** 2025-12-05

**Authors:** M. Isabela Troya, Ella Arensman, Robert Briggs, Eve Griffin, Caoimhe Lonergan, Sally Ann Lovejoy, Faraz Mughal, James O Mahony, Mark Ward

**Affiliations:** 1 School of Public Health, College of Medicine and Health, University College Cork, Cork, Ireland; 2 National Suicide Research Foundation, University College Cork, Cork, Ireland; 3 Australian Institute for Suicide Research and Prevention, School of Applied Psychology, Griffith University, Brisbane, Queensland, Australia; 4 The Irish Longitudinal Study on Ageing, Trinity College Dublin, Dublin, Ireland; 5 Department of Psychiatry and Neurobehavioral Science, University College Cork, Acute Mental Health Unit, Cork University Hospital, Wilton, Ireland; 6 National Clinical Programme for Self-Harm and Suicide-related Ideation, Office of the National Clinical Advisor and Group Lead, Dr. Steevens Hospital, Dublin, Ireland; 7 School of Medicine, Keele University, Keele, United Kingdom; 8 School of Nursing & Midwifery, University College Cork, Cork, Ireland

**Keywords:** Wish to die, Death ideation, Healthcare use, Older adults, Primary care

## Abstract

**Aim:**

To examine the relationship between wish to die (WTD) and healthcare use in primary and secondary care among older adults living in Ireland.

**Subject and methods:**

Secondary analysis of a nationally representative sample of community-dwelling older adults from The Irish Longitudinal Study on Ageing (TILDA). Primary outcomes were self-reported general practitioner (GP) and emergency department (ED) visits in the last 12 months. Negative binomial regression was used to examine the associations between WTD and healthcare use.

**Results:**

Out of 8174 individuals aged 50 and older, a total of 8149 provided information relating to WTD at wave 1. Of these, 279 (3.4%) individuals disclosed a WTD, while 7870 did not. The mean number of self-reported GP visits in the last 12 months was 4.81 (standard error [SE] = 0.29, 95% confidence interval [CI] 4.25–5.37) for those disclosing a WTD (*n* = 261), while it was 3.59 (SE = 0.04, 95% CI 3.51–3.67) for those without a WTD (*n* = 7791). WTD was associated with a higher number of GP visits (incidence rate ratio [IRR] = 1.03, *p* = 0.03, 95% CI 1.00–1.06). After adjusting for relevant covariates, associations between WTD and ED visits were not observed. Female gender, lower education levels, living alone, depressive and anxiety symptoms, chronic health conditions, severe chronic pain, problematic alcohol consumption, smoking, falls, disability, and regular medication use showed significant associations with WTD. Limitations of the study include the presentation of cross-sectional results; thus we do not make causal inferences or conclusions on the directionality/trajectory of WTD and healthcare use.

**Conclusion:**

Older adults reporting WTD presented to GPs approximately five times within the previous 12 months. While effect sizes were small, these findings may help inform health services. Future research can further examine potential longitudinal associations and whether GP and ED attendance patterns change over time.

## Introduction

Older adults represent 10% of the population globally and are the age group with the greatest predicted growth in future years (United Nations 2024). With the existing changes in population demographics, healthcare systems must adapt to increasingly ageing populations and cater to the healthcare needs of older adults (Jones and Dolsten 2024). Older adults have more complex healthcare needs than younger age groups, due to frailty and comorbid health conditions, with previous research finding that older adults have increased healthcare utilisation compared to younger populations (Adams et al. 2015; Adepoju et al. 2018; Cheng et al. 2020; Roe et al. 2017).

International research shows that mental health problems such as depressive symptoms are associated with increased healthcare use, including a higher risk of hospitalisation, longer length of stay, and a higher re-admission risk in adults and older adults (Buczak-Stec et al. 2022; Luppa et al. 2012; Prina et al. 2015). Similarly, there is evidence of increased healthcare use amongst adults and older adults with anxiety (Hohls et al. 2018; Horenstein and Heimberg 2020) and older adults experiencing loneliness, a known risk factor for mental health problems (Burns et al. 2020). However, some research reports that older adults are reluctant to seek support for their mental health problems due to stigma and shame (Gonçalves et al. 2014; Lavingia et al. 2020). While shame and stigma often preclude formal help-seeking for self-harm (Troya et al. 2019a), older adults are increasingly accessing healthcare services for support with self-harm and suicide-related ideation (Morgan et al. 2018; Schmutte et al. 2022).

Physical health conditions, in particular chronic and life-threatening health conditions, have been associated with an increased wish to die and suicidality amongst individuals, including older adults (Ernst et al. 2024; Lapierre et al. 2015; Ohnsorge et al. 2014; Rogers et al. 2021). Given that people with chronic health conditions often have regular healthcare appointments to manage their conditions (Rogers et al. 2021), it is important to consider and explore the impact of physical and chronic health conditions on wish to die and healthcare use, in particular in older age groups.

Wish to die (WTD), defined as the feeling that one would be better off dead or wishing for one’s death (Bornet et al. 2020), is often referred to as the first step in the suicidal process, which could ultimately lead to future suicidal thoughts and behaviours (Harmer et al. 2023). WTD may also involve suicidal thoughts (Harmer et al. 2023). Across the main psychological theories of suicide, including the interpersonal theory of suicide (Joiner 2005) and the integrated motivational–volitional (IMV) model (O’Connor and Kirtley 2018), death wishes represent a starting point on the pathway to suicidal behaviour (Klonsky et al. 2018). For instance, in the IMV model, WTD is a sign of entrapment and defeat in the motivational phase, and in Joiner’s interpersonal theory of suicide, WTD emerges before acquired capability, as a desire for death (Joiner 2005; O’Connor and Kirtley 2018). WTD is positively associated with depression in older adults (Bornet et al. 2020) and is an important clinical indicator for future self-harm, mental health problems, and suicide (Harmer et al. 2023; Lapierre et al. 2015).

Early detection and intervention are key in supporting mental health and addressing suicide-related thoughts and behaviours; therefore, it is imperative to know whether older adults who experience WTD access healthcare services frequently. In particular, non-specialised mental health settings such as primary care can be an important setting for detection of WTD. To the best of our knowledge, there is no research examining the relationship between WTD and healthcare use in older adults. The aim of this study was to examine the association between WTD and healthcare use in primary care (number of general practitioner [GP] visits) and secondary care (number of emergency department [ED] visits) in a nationally representative cohort of community-dwelling older adults in Ireland. We hypothesised that WTD would be associated with increased healthcare use in older adults; however, relevant healthcare conditions may lead to this increase.

## Methods

### Study design and population

This was a cross-sectional study using wave 1 of The Irish Longitudinal Study on Ageing (TILDA). TILDA is a largescale nationally representative longitudinal cohort study of community-dwelling adults aged 50 years and over, using multi-stage, stratified random sampling from 640 geographical areas in Ireland. Clustered random sampling was used to obtain nationally representative samples. Institutionalised older adults, individuals with dementia, and individuals unable to provide written informed consent due to severe cognitive impairment were not eligible for inclusion in the study. A total of 8175 computer-assisted personal interviews (CAPI) were conducted by trained personnel at baseline, with a response rate of 62%. Among these respondents, 85% (*n* = 6915) also returned completed self-completion questionnaires (SCQs). Full details of the TILDA survey including sampling methods and recruitment have been further described elsewhere (Donoghue et al. 2018). As this was an initial exploratory study, we focused on one time point (wave 1, data collected between October 2009 and February 2011), and future studies may further examine longitudinal associations. TILDA obtained ethical approval from the Faculty of Health Sciences Research Ethics Committee at Trinity College Dublin. Written informed consent was provided by all participants.

### Exposure variable

Wish to die was the main exposure variable. Participants who reported WTD were identified through self-report. WTD was captured using the single (yes/no) question from the EURO-D Scale: ‘In the last month have you felt you would rather be dead?’ (Prince et al. 1999). WTD was measured using the CAPI questionnaire.

### Outcomes

The primary outcome of this study was self-reported healthcare use in the previous 12 months. This included the number of primary care (GP) and secondary care (ED) visits in the last 12 months. Healthcare use was measured using the CAPI questionnaire.

### Covariates

On the basis of existing literature (Bonnewyn et al. 2017; Briggs et al. 2021; Jorm et al. 1995), we identified the following relevant covariates to include in our study: self-reported doctor-diagnosed chronic conditions (0, 1, 2, or 3 or more), smoking status (never, past, current), problematic alcohol consumption (yes/no) measured using the CAGE questionnaire (Mayfield et al. 1974), chronic pain (no pain, mild, moderate, severe), number of self-reported regular medications (0, 1–2, 3–4, or 5 or more), falls in the last year (none, 1 or more). Depressive symptoms were measured using the eight-item version of the Center for Epidemiological Studies Depression (CES-D) scale (Radloff 1977). Scores ranged from 0 to 24, with a score of 9 or more defined as clinically significant depression symptoms (Briggs et al. 2018a). Anxiety symptoms were measured using the subscale of the Hospital Anxiety and Depression Scale (Zigmond and Snaith 1983). Anxiety scores ranged from 0 to 21, with scores of 8 or more defined as a positive screen for general anxiety disorder (GAD), consistent with previous studies (Bjelland et al. 2002; Olssøn et al. 2005; Smith et al. 2022). We also controlled for the presence of cardiovascular (CVD) conditions (yes/no) and disability status using the activities of daily living (ADL) and instrumental activities of daily living (IADL) measures (not disabled, IADL limitation only, ADL limitation only, both) (Kojima 2018). All the covariates identified were measured using the CAPI questionnaire, with the exception of anxiety symptoms and problematic alcoholic consumption, which were captured using the SCQs.

### Statistical analyses

First, relevant variables and sociodemographic characteristics were identified and compared with those reporting WTD using the *t*-test and chi-square statistics as appropriate. A negative binomial model was estimated as the outcome variables (GP/ED visits) were zero-inflated and over-dispersed, meaning that the variance was greater than the mean (Sarma and Simpson 2006). When calculated, the dispersion parameter α confirmed that the data were over-dispersed (model α > 0). Three negative binomial models were estimated: (a) Model 1, unadjusted; (b) Model 2, adjusted for medical card (free public healthcare) and/or private health insurance status; and (c) Model 3, adjusted for covariates and sociodemographic variables. Given the varying samples between models 1 and 3, we conducted a sensitivity analysis to examine the relationship between healthcare use and WTD with the same sample size. Incident rate ratios (IRRs) with 95% confidence intervals (CI) are used to present the findings, and a *p* value of less than 0.05 is considered statistically significant. Stata version 15 (StataCorp 2017) software was used to perform the analysis.

## Results

Out of 8174 individuals aged 50 and older, a total of 8149 provided information relating to WTD at wave 1. Of these, 279 (3.4%) individuals disclosed a WTD, while 7870 did not. Table 1 provides the main sociodemographic, health, and healthcare use characteristics of individuals based on WTD. Female gender, younger age, lower levels of education (none/primary vs secondary or higher), living alone (as opposed to living with spouse or others), marital status of separated, divorced or widowed (as opposed to married), severe depressive symptoms, anxiety symptoms, three or more chronic health conditions (as opposed to two or fewer), severe chronic pain, problematic alcohol consumption, current smoking, falls within the last year, any type of disability, and the use of five or more regular medications showed significant associations with WTD (see Table 1).

The mean number of self-reported GP visits in the last 12 months was 4.81 (standard error [SE] = 0.29, 95% CI 4.25–5.37) for those disclosing a WTD (*n* = 261), while it was 3.59 (SE = 0.04, 95% CI 3.51–3.67) for those without a WTD (*n* = 7791), resulting in a mean difference of 1.22 presentations between the two groups (*t* = 5.44, *p* = 0.001, 95% CI 0.78–1.66). The number of presentations to the GP within the last 12 months varied from 0 to 150. The number of presentations to the ED in the last 12 months varied from 0 to 50. The mean number of ED visits for those disclosing a WTD (*n* = 279) was 0.24 (SE = 0.38, 95% CI 0.50–0.99), while for those without a WTD (*n* = 7870), it was 0.16 (SE = 0.03, 95% CI 0.10–0.23) (*t* = 0.40, *p* = 0.70, 95% CI 0.29–0.44).

WTD was significantly associated with increased GP visits when adjusting for all covariates, as seen in Table 2, Model 3. However, the effect size was small (1.03, 95% CI 1.00–1.06, *p* = 0.03). When examining ED presentations in older adults reporting WTD, the unadjusted (Model 1) and health insurance-adjusted (Model 2) models found reduced healthcare use (see Table 2). No significant associations were observed in the fully adjusted (Model 3) model for WTD and ED visits.

Older adults reporting moderate and severe depressive symptoms had increased GP visits compared to those reporting mild depressive symptoms; these findings were not replicated in ED visits (see Table 2, Model 3). Reporting of anxiety symptoms of any severity resulted in increased GP visits among older adults when compared to those who did not report any type of anxiety symptoms. Increased ED visits were found in those with severe anxiety symptoms compared to those with no anxiety symptoms. With regard to disability, there was evidence of increased GP and ED visits for those with IADL and ADL disability. Older adults reporting one or more chronic health conditions were more likely to visit their GPs than those who did not have any chronic health conditions. Older adults taking regular medication had increased GP and ED visits when compared to those who did not report consuming regular medication. Chronic pain was also associated with increased GP and ED visits relative to those who did not report chronic pain. Older people reporting a cardiovascular condition had more ED visits than those who did not report any cardiovascular conditions. There were fewer ED visits in older people who reported one or more falls in the past year than in those who did not. Older adults who had a tertiary level or higher education status reported a greater number of ED visits than those with up to primary0level education, while older adults who were separated or divorced reported increased GP visits compared to those who were married. In relation to medical insurance/coverage, Models 2 and 3 provide evidence that older people with insurance accessed primary care more often than those who did not have coverage. In contrast, visits to EDs for those who had medical insurance/coverage were reduced compared to older people with no insurance coverage (see Table 2).

### Sensitivity analysis

As observed in Supplementary Table 1, findings from the sensitivity analysis, where the same number of participants were used, are congruent with the previous models, showing reduced GP and ED visits in Models 1 and 2.

## Discussion

Using data from a large, nationally representative cohort of community-dwelling older adults, we found that older adults with WTD disclosed visiting their GP multiple times a year, while ED presentations were not as common and were reported only in a subset of older adults. After adjusting for relevant sociodemographic and health-related variables, small but significant effect sizes were observed of increased presentations to GPs amongst older adults reporting WTD (IRR 1.03, *p* = 0.03). We identified relevant sociodemographic and health-related variables for increased healthcare use, most notably elevated GP visit numbers among participants with depressive symptoms, anxiety symptoms, chronic health conditions, chronic pain, disability, and regular medication use. No significant associations were found between healthcare use in ED settings and older adults disclosing WTD in the adjusted model (Model 3). Increased ED visits were reported among older adults with depressive symptoms, anxiety symptoms, chronic pain, cardiovascular conditions, disability, and regular medication use.

Older adults are at increased risk of developing chronic health conditions when compared to younger people (Adams et al. 2015). On the basis of existing literature (Bonnewyn et al. 2017; Briggs et al. 2021; Jorm et al. 1995), we identified relevant (physical and mental) health-related covariates in our analysis as described in the methods section. While previous research has shown that mental health problems such as depressive symptoms (Luppa et al. 2012; Prina et al. 2015), anxiety (Horenstein and Heimberg 2020), and loneliness (Burns et al. 2020) result in increased healthcare use among older adults, no previous research had examined the relationship between WTD and healthcare use. Our findings identified physical health-related variables (including chronic health conditions, chronic pain, disability) with increased healthcare use. The role of physical health, particularly in older age, should be considered when thinking about WTD amongst individuals (Rogers et al. 2021). As argued by Rogers et al. (2021), there is an opportunity to provide continued care and support for individuals with chronic health conditions who report suicidality or WTD.

Despite international research reporting increased healthcare use in ED settings amongst older adults (Samaras et al. 2010), our findings showed that ED presentations were rare among our sample, and no increased ED presentations were observed in older adults reporting WTD. In contrast, there was evidence of reduced ED presentations amongst older adults reporting WTD, although these findings were not repeated in the multivariate model. Furthermore, we cannot know whether WTD determined the frequency of ED or GP presentations, given our study design. Our previous qualitative research conducted in Ireland examined barriers older adults with suicidal behaviour experience when seeking support in EDs, with participants reporting the ED being an unsuitable environment, with excessive waiting times and unclear referral pathways (Troya et al. 2024).

As WTD can often, but not always, be the first step for future suicidal behaviours, and may overlap with suicidal thoughts, our research provides important insights for suicide prevention. Although WTD and healthcare use were assessed at different time points and we cannot determine causality, our research shows that older adults are in frequent contact with their GPs, with an estimated 4.81 visits per year for those disclosing WTD and 3.59 for those without WTD. Existing literature highlights the opportunity for prevention and early intervention in future suicidal behaviour in older adults within a primary care setting (Mughal et al. 2020). Primary care has been identified as an optimal health system to prevent suicidal behaviour in older adults, as evidenced by previous systematic reviews (Laflamme et al. 2022; Okolie et al. 2017). Previous qualitative research conducted in Ireland identified that GPs demonstrate positive attitudes towards patients presenting with self-harm (O’Donohoe 2022). Furthermore, previous qualitative research has also identified primary care as an important setting for supporting older adults with suicidal behaviour given older adults’ frequent contact with GPs due to comorbid health conditions (Troya et al. 2024; Wand et al. 2018; Frost et al. 2019). Existing research has shown that late-life depression is often undiagnosed and untreated amongst older adults, with up to two thirds of Irish older adults reporting symptoms of depression not receiving any treatment (Briggs et al. 2018b). Given the reported associations between depression (Briggs et al. 2021), self-harm (Troya et al. 2019b), suicide (Conejero et al. 2018), and WTD amongst older adults, adequate identification, management, support, and treatment of late-life depression is needed to prevent future escalation of thoughts and behaviours.

### Strengths and limitations

We report findings based on a large, nationally representative sample of community-dwelling older adults living in Ireland. Our findings must be interpreted with caution, as we present cross-sectional results from the first wave of data from TILDA providing initial evidence of a potential association between WTD and healthcare use. Participants were asked about reports of WTD in the last month, while healthcare use was in the last 12 months, constituting different time points. Our findings do not permit causal inferences or conclusions on directionality or trajectory of WTD and healthcare use. Future research may explore longitudinal associations between WTD and healthcare use to further our understanding on how WTD impacts subsequent healthcare use. Furthermore, given the relationship between WTD and further suicidal thoughts and behaviours, it would have been useful to assess other suicidal thoughts or behaviours. However, TILDA only collected data on WTD, and no other relevant suicidal outcomes were included. WTD was measured using the single (yes/no) question from the EURO-D Scale: ‘In the last month have you felt you would rather be dead?’ (Prince et al. 1999). While other research (Briggs et al. 2021; Ward et al. 2024) has used the same item to assess WTD, and the predictive validity of the EURO-D scale has been validated with the TILDA cohort (Briggs et al. 2018a), we did not use a specific instrument or questionnaire to measure WTD (such as the Schedule of Attitudes Toward Hastened Death or the Categories of Attitudes Toward Death Occurrence), but instead relied on the available data from the study. This limitation should be considered when interpreting findings from our study.

Given the stigma associated with WTD and suicide-related thoughts and behaviours, especially amongst older adults, it is possible that death wishes were under-reported in this study. Furthermore, healthcare use information was provided based on self-report of participants, and therefore it is subject to recall bias. Given that participants responded regarding their healthcare use in the last 12 months, this number is likely an underestimate.

## Conclusion

To the best of our knowledge, findings from our research are the first to examine associations between WTD and healthcare use. Older adults reporting a WTD visit their GP approximately five times a year (1.22 visits more than those without WTD), while ED visits are rarer amongst older adults with (0.24 mean visits) and those without (0.16 mean visits) WTD. We observed small effect sizes for an increase in GP visits amongst those reporting WTD. These findings add to the existing literature which highlights that health practitioners, in particular GPs, are well placed to detect and support older adults reporting death wishes and are positioned to provide management and treatment to avoid suicide-related thoughts and behaviours, in particular among older adults reporting depressive and anxiety symptoms, chronic pain, disability, and chronic health conditions. Future research can further examine potential longitudinal associations and whether GP and ED attendance patterns change over time.

## Supplementary Material

The online version contains supplementary material available at https://doi.org/10.1007/s10389-025-02647-2

Supplementary Material

## Figures and Tables

**Table 1:** Sociodemographic and health characteristics by WTD wave 1 TILDA (*n* = 8149)

	WTH (*n* = 279) (3.41%)	No WTH (*n* = 7870) (96.59%)	*t/*χ^2^(*p* value)
Gender (*n* = 8149)			
Male	111 (2.97%)	3626 (97.03%)	4.29 (*p* < 0.05)
Female	168 (3.81%)	4244 (96.19%)	
Age, mean (SE, SD) (*n* = 8148)	62.3 (0.60, 9.8)	63.9 (0.11, 9.7)	–2.66 (*p* < 0.01)
Education (*n* = 8145)			
Primary/none	120 (4.81%)	2377 (95.19%)	21.38 (*p* < 0.01)
Secondary	94 (2.89%)	3159 (97.11%)	
Tertiary/higher	64 (2.67%)	2331 (97.33%)	
Living conditions (*n* = 8149)			
Alone	93 (5.14%)	1718 (94.86%)	24.87 (*p* < 0.001)
With spouse	80 (2.47%)	3154 (97.53%)	
With others	106 (3.41%)	2998 (96.59%)	
Marital status (*n* = 8149)			
Married	156 (2.77%)	5469 (97.23%)	51.79 (*p* < 0.001)
Never married	24 (3.06%)	761 (96.94%)	
Separated/divorced	46 (8.35%)	505 (91.65%)	
Widowed	53 (4.46%)	1135 (95.54%)	
Healthcare use			
GP visits, mean (SE, SD) (*n* = 8052)	4.81 (0.29, 4.64)	3.63 (0.04, 3.51)	5.44 (*p* < 0.001)
ED visits, mean (SE, SD) (*n* = 8147)	0.24 (0.38, 6.4)	0.17 (0.03, 2.93)	0.40 (*p* = 0.70)
CES-D–depressive symptoms (*n* = 8029)			
None/mild (0–7)	51 (0.87%)	5797 (99.13%)	826.6 (*p* < 0.001)
Moderate (8–15)	56 (3.97%)	1356 (96.03%)	
Severe (16+)	158 (20.55%)	611 (79.45%)	
HADS–anxiety symptoms (*n* = 6615)			
None (0–6)	72 (1.44%)	4939 (98.56%)	327.9 (*p* < 0.001)
Mild (8–10)	56 (5.5%)	963 (94.5%)	
Moderate (11–15)	58 (11.79%)	434 (88.21%)	
Severe (16+)	23 (24.73%)	70 (75.27%)	
Chronic conditions (*n* = 8149)			
0	41 (2.23%)	1793 (97.77%)	30.97 (*p* < 0.001)
1	63 (2.76%)	2218 (97.24%)	
2	64 (3.38%)	1828 (96.62%)	
3 or more	111 (5.18%)	2029 (94.82%)	
Chronic pain (*n* = 7859)			
None	110 (2.09%)	5152 (97.91%)	187.5 (*p* < 0.001)
Mild	33 (3.99%)	794 (96.01%)	
Moderate	51 (3.79%)	1293 (96.21%)	
Severe	85 (12.02%)	622 (87.98%)	
Cardiovascular condition (*n* = 8149)			
Yes	78 (2.67%)	2845 (97.33%)	7.85 (*p* < 0.001)
No	201 (3.85%)	5025 (96.15%)	
Alcohol problem (*n* = 6739)			
Yes	54 (6.65%)	758 (93.35%)	36.25 (*p* < 0.001)
No	160 (2.70%)	5767 (97.30%)	
Smoking status (*n =* 8148)			
Never	91 (2.56%)	3463 (97.44%)	41.63 (*p* < 0.001)
Past	97 (3.12%)	3011 (96.88%)	
Current	91 (6.12%)	1395 (93.88%)	
Fall(s) past year (*n* = 8147)			
Yes	88 (5.59%)	1487 (94.41%)	27.69 (*p* < 0.001)
No	191 (2.91%)	6381 (97.09%)	
Disability (*n* = 8149)			
None	186 (2.59%)	6984 (97.41%)	176.1 (*p* < 0.001)
IADL only	33 (11.50%)	254 (88.5%)	
ADL only	17 (4.43%)	367 (95.57%)	
Both	43 (13.96%)	265 (86.04%)	
No. of regular medications (*n* = 8067)			
0	53 (2.32%)	2233 (97.68%)	52.86 (*p* < 0.001)
1–2	53 (2.20%)	2359 (97.80%)	
3–4	65 (3.83%)	1633 (96.17%)	
≥ 5	99 (5.92%)	1572 (94.08%)	

*ADL* activities of daily living, *CES-D* Center for Epidemiologic Studies Depression Scale, *HADS* Hospital Anxiety and Depression Scale, *IADL* instrumental activities of daily living, *SD* standard deviation, *SE* standard error

**Table 2:** Negative binomial regression estimating the association between healthcare use and WTD, wave 1 TILDA Models 1–3

(a) Model 1: Unadjusted				
	**GP visits** (*n* = 8042) IRR (95% CI)	*p* value	**ED visits** (*n* = 8142) IRR (95% CI)	*p* value
WTD (Ref)	0.93 (0.90–0.96)	0.001	0.80 (0.73–0.87)	0.001
(b) Model 2: Adjusted for insurance coverage				
	**GP visits** (*n* = 8042) IRR (95% CI)	*p* value	**ED visits** (*n* = 8136) IRR (95% CI)	*p* value
WTD (Ref)	0.94 (0.92–0.97)	0.001	0.84 (0.78–0.92)	0.001
Insurance, none (Ref)				
Medical insurance only	1.13 (1.06–1.22)	0.001	0.74 (0.58–0.93)	0.01
Medical card only	2.26 (2.10–2.43)	0.001	1.16 (0.92–1.48)	0.20
Dual coverage	2.06 (1.90–2.22)	0.001	1.28 (0.99–1.65)	0.06
(c) Model 3: Multivariate model				
	**GP visits** (*n* = 6291) IRR (95% CI)	*p* value	**ED visits** (*n* = 6350) IRR (95% CI)	*p* value
WTD (Ref)	1.03 (1.00–1.06)	0.03	1.00 (0.89–1.12)	0.95
Male (Ref)	0.98 (0.94–1.02)	0.33	0.94 (0.81–1.11)	0.50
Primary education (Ref)				
Secondary	0.99 (0.94–1.04)	0.72	1.00 (0.82–1.22)	0.97
Tertiary/higher	0.95 (0.90–1.00)	0.07	1.30 (1.03–1.63)	0.02
Lives alone (Ref)				
Lives with spouse	1.03 (0.94–1.13)	0.48	1.03 (0.72–1.48)	0.84
Lives with others	1.00 (0.93–1.08)	0.94	1.18 (0.88–1.60)	0.26
Married (Ref)				
Never married	1.10 (1.00–1.21)	0.06	1.44 (1.02–2.06)	0.04
Separated/divorced	1.11 (1.00–1.22)	0.05	1.01 (0.69–1.50)	0.95
Widowed	1.04 (0.95–1.13)	0.43	0.93 (0.66–1.34)	0.72
Insurance, none (Ref)				
Medical insurance only	1.09 (1.01–1.18)	0.03	0.74 (0.56–0.98)	0.03
Medical card only	1.62 (1.49–1.75)	0.001	1.00 (0.75–1.35)	0.98
Dual coverage	1.47 (1.35–1.60)	0.001	1.07 (0.79–1.46)	0.67
Depressive symptoms, mild (Ref)				
Moderate	1.12 (1.07–1.18)	0.001	1.07 (0.87–1.31)	0.53
Severe	1.22 (1.13–1.32)	0.001	1.14 (0.84–1.54)	0.41
Anxiety symptoms, none (Ref)				
Mild	1.07 (1.01–1.13)	0.02	1.05 (0.84–1.30)	0.69
Moderate	1.11 (1.02–1.20)	0.01	1.07 (0.78–1.46)	0.69
Severe	1.25 (1.07–1.46)	0.001	2.26 (1.28–4.01)	0.01
Chronic health conditions, 0 (Ref)				
1	1.14 (1.07–1.22)	0.001	1.18 (0.91–1.53)	0.20
2	1.19 (1.10–1.28)	0.001	0.80 (0.59–1.09)	0.16
3 or more	1.27 (1.17–1.39)	0.001	1.17 (0.84–1.62)	0.36
Chronic pain, none (Ref)				
Mild	1.00 (0.94–1.07)	0.93	1.37 (1.07–1.75)	0.01
Moderate	1.23 (1.17–1.30)	0.001	1.19 (0.96–1.47)	0.12
Severe	1.38 (1.29–1.49)	0.001	1.37 (1.03–1.82)	0.03
Cardiovascular condition (Ref)	1.02 (0.96–1.08)	0.55	1.26 (1.00–1.57)	0.04
Alcohol problem, no (Ref)	0.94 (0.88–1.00)	0.05	1.04 (0.82–0.90)	0.73
Yes				
Smoker, never (Ref)				
Past	1.00 (0.96–1.04)	0.87	0.98 (0.82–1.16)	0.78
Current	0.94 (0.89–1.00)	0.05	0.86 (0.69–1.08)	0.21
Fall(s) past year (Ref)	0.99 (0.97–1.00)	0.01	0.86 (0.82–0.90)	0.001
Disability, none (Ref)				
IADL only	1.11 (1.00–1.24)	0.04	1.47 (0.99–2.17)	0.05
ADL only	1.10 (1.00–1.19)	0.04	1.12 (0.80–1.58)	0.51
Both	1.09 (0.98–1.20)	0.09	1.51 (1.02–2.21)	0.04
Regular medications, 0 (Ref)				
1–2	1.81 (1.70–1.92)	0.001	1.41 (1.11–1.79)	0.004
3–4	2.12 (1.98–2.28)	0.001	2.51 (1.93–3.28)	0.001
≥ 5	2.43 (2.26–2.62)	0.001	2.49 (1.85–3.35)	0.001

## Data Availability

The public TILDA dataset supporting the conclusions of this article is available from the Irish Social Science Data Archive (ISSDA) University College Dublin, which can be accessed here: http://www.ucd.ie/issda/data/tilda/
