# Supplementary material for: Wish to die and healthcare use in older people: cross-sectional findings from The Irish Longitudinal Study on Ageing (TILDA)
Source: J Public Health (Berl). Author manuscript; Available in PMC 2026 Jan 6. (PMC7618591; doi:10.1007/s10389-025-02647-2)
Supplement: Supplementary Material [file EMS211894-supplement-Supplementary_Material.docx]

Supplementary Table 1. Sensitivity analysis Negative binomial regression estimating the association between healthcare use and WTD Wave 1 TILDA Models 1-2

|  | No. | Model 1  IRR (95% CI), *p* value | No. | Model 2  IRR (95% CI), *p* value |
| --- | --- | --- | --- | --- |
| GP visits  ED visits | 6291  6350 | 0.94 (0.91-0.97), *p*<0.001  0.87 (0.78-0.97), *p=*0.01 | 6291  6350 | 0.96 (0.93-0.99), *p=*0.01  0.90 (0.81-1.00), *p=*0.05 |

^IRR= Incident Rate Ratio; CI= Confidence Interval^
^Model 1 unadjusted. Model 2 adjusted for insurance cover.^
